# Stagewise resolution of temperature-dependent embryonic and postembryonic development in the cowpea seed beetle *Callosobruchus maculatus* (F.)

**DOI:** 10.1186/s12898-020-00318-2

**Published:** 2020-09-11

**Authors:** Dmitry Kutcherov

**Affiliations:** Department of Entomology, St. Petersburg State University, St. Petersburg, 199034 Russia

**Keywords:** *Callosobruchus maculatus*, Development, Embryo, Lower temperature threshold, Plasticity, Reaction norm, Seed beetles, Sum of degree-days, Temperature

## Abstract

**Background:**

The thermal plasticity of life-history traits receives wide attention in the recent biological literature. Of all the temperature-dependent traits studied, developmental rates of ectotherms are especially often addressed, and yet surprisingly little is known about embryonic responses to temperature, including changes in the thermal thresholds and thermal sensitivity during early development. Even postembryonic development of many cryptically living species is understood superficially at best.

**Results:**

This study is the first to estimate the exact durations of developmental stages in the cowpea seed beetle *C. maculatus* from oviposition to adult emergence at five permissive constant temperatures from 20 to 32 °C. Early embryonic development was tracked and documented by means of destructive sampling and subsequent confocal imaging of fluorescently stained specimens. Late embryonic and early larval development was studied with the use of destructive sampling and light microscopy. Well-resolved temporal series based on thousands of embryos allowed precise timing of the following developmental events: formation of the blastoderm; formation, elongation, and retraction of the germ band; dorsal closure; the onset and completion of sclerotization of the cuticle; hatching, and penetration of the first-instar larva into the cowpea seed. Pupation and adult eclosion were observed directly through an incision in the seed coat. The thermal phenotype of *C. maculatus* was found to vary in the course of ontogeny and different stages scaled disproportionately with temperature, but pitfalls and caveats associated with analyses of relative durations of individual stages are also briefly discussed.

**Conclusion:**

Disproportionate changes in developmental durations with temperature may have important implications when study design requires a high degree of synchronization among experimental embryos or when the occurrence of particular stages in the field is of interest, as well as in any other cases when development times need to be estimated with precision. This work provides one of the first examples of integration of embryological techniques with ecophysiological concepts and will hopefully motivate similar projects in the future. While experiments with *Drosophila* continue to be the main source of information on animal development, knowledge on other model species is instrumental to building a broader picture of developmental phenomena.

## Background

Organismal responses to temperature have become a popular object of eco-evolutionary research in recent years. This surge in scientific interest was in part driven by the globally observed climate warming [48], but, besides that, a growing number of biologists are starting to appreciate the important and yet commonly underrated role played by phenotypic plasticity in adaptive evolution [71]. This is why more and more studies address not only mean trait values but also the degree of trait sensitivity to various ecological factors, including temperature, and many familiar biological terms are reiterated with an epithet ‘thermal’: thermal reaction norm [2], thermal coevolution [4], thermal transcriptome [63], etc. Such studies are important both for our understanding of the molding of life histories in the evolutionary past and for predicting the responses of populations and communities to the consequences of human activity in the future [1].

Recently, a promising branch of life sciences has formed at the confluence of developmental and evolutionary biology: the so-called evo-devo, which aims to provide an answer to the central question of why living organisms look and operate the way they do [20]. Perhaps unsurprisingly, the term ‘thermo-evo-devo’ has already been coined as well [70]. However, despite a multitude of topics in common, thermal ecology and evo-devo rarely meet under the same article title. While ecologists view various temperature effects on development as one of the mainstream avenues of research, developmental biologists are very seldom interested in how development proceeds at temperatures higher or lower than the standard regime [72], one major exception being the studies of temperature-sensitive genes [49]. Meanwhile, from an ecologist’s point of view, development is often merely a period from one conspicuous transformation to the other with all the events in-between being irrelative to the main purpose of the work. This is especially true of studies that deal with temperature-dependent animal embryogenesis and largely dismiss everything that occurs between oviposition and hatching [11, 22, 37, 40] (see also a review by Howe [24], which remains the only comprehensive treatise of this subject in insects so far). Examples of integration of thermal developmental ecology with embryology, or vice versa, also do exist, yet these rare studies mostly deal with animals whose embryos, or at least certain embryonic stages, are more or less easily observable in vivo [10, 34, 46, 55, 56, 61]. Still, even in model species, very little is known about the thermal phenotype during early development and the changes in this phenotype from one stage to the other.

One of the most commonly studied temperature-dependent developmental traits is durations of various stages and the reciprocals of durations, i.e., developmental rates, which are important in modeling phenology and population dynamics and are relatively easy to measure. Such experiments typically involve a set of environmental chambers with constant temperatures where development is monitored at regular intervals of time (most often daily). In contrast to many other temperature responses, the shapes of which can only be tentatively imagined prior to the experiment, the shape of the thermal reaction norm for developmental rate is rather conservative and has been thoroughly analyzed, at least in plants and ectothermic invertebrates [13, 27, 64]. Over the entire range of developmental temperatures, this reaction norm is best approximated by an asymmetrical bell-shaped curve, but, in the permissive (favorable) temperature range, developmental rate increases with temperature in a quasi-linear fashion [6, 27]. Unless one is specifically interested in thermal stress, this linear model is sufficiently precise for most purposes and has been applied to a vast number of economically important species [26, 35, 45].

Seed beetles (Coleoptera: Chrysomelidae: Bruchinae) that develop in stored pulses are one example of agricultural pests whose developmental responses to temperature are relatively well studied [31, 32, 60, 67]. These beetles spend most of their life cryptically, inside dry seeds, and so their whole development at a given temperature is usually described at the coarsest possible resolution, i.e., from oviposition to adult emergence. Notable exceptions include a study of temperature-dependent development in *Callosobruchus rhodesianus* (Pic) with the aid of X-ray photography [25] and research on *Bruchus pisorum* (L.) where careful examination of eggs [58] and destructive sampling of postembryonic stages [59] provide a substantially better understanding of the effects of temperature during ontogeny. By far the greatest number of articles are devoted to the temperature-dependent development in the cowpea seed beetle *Callosobruchus maculatus* (F.), either exclusively or alongside other Bruchinae and using various legumes as hosts [8, 9, 18, 19, 25, 36, 42, 47, 62, 66, 67]. All these studies but three [9, 19, 47], to which I will return below, only report oviposition-to-adult-emergence periods. While this level of detail may be sufficient for purely practical purposes, describing the thermal phenotype in such broad-brush terms arguably does not do justice to the diverse and intricate processes responsible for the transformation of an inseminated oocyte into a mature holometabolous insect. Also, insights into the evolutionary pathways that have led to the specialization of Bruchinae on seeds would benefit from a deeper understanding of how at least one better studied member of this subfamily comes into being in the course of ontogeny.

Bruchinae, with approximately 1650 recent species [43], are one of the largest lineages of insects to have colonized the sheltered and nutrient-rich microhabitats found inside plant seeds. Apart from the obvious change in the trophic niche, the ancestors of modern seed beetles inevitably had to adapt to a novel thermal niche as, e.g., many forms of thermoregulation were no longer an option for a white, short-legged or legless larva enclosed inside an immovable seed. Another, much more recent, change in the thermal niche occurred when some seed beetles colonized human stores of pulses worldwide and so became buffered from strong fluctuations of temperature for multiple generations. Further still, *C. maculatus* made its way into laboratories where it is widely used as a model organism in diverse areas of biology [30] and its stock cultures continue to thrive in stable thermal conditions.

This study was motivated by the paucity of stage-resolved data on immature development in *C. maculatus* and is an endeavor to fill this gap from the perspective of thermal ecology, yet with an emphasis on embryogenesis. Some guide figures on the timing of consecutive cleavages in *C. maculatus* at three incubation temperatures are already available [68] but hardly sufficient for plotting thermal reaction norms. Another study [14] briefly reports on later embryonic development at a single temperature of 28 °C. Regarding the postembryonic development in this beetle, two studies [19, 47] relied on external changes and did not directly observe neither hatching, nor pupation, nor eclosion, while the third one [9] unfortunately lacks precision, even though the experimental design involved daily dissection of seeds.

Here, development in *C. maculatus* is tracked at five constant temperatures ranging from 20 to 32 °C. Early development until dorsal closure is studied by fixation and fluorescent staining of groups of embryos at different times from egg-laying. Later development is studied by dissection and visual inspection of groups of embryos and first-instar larvae. Since both techniques imply destructive sampling, logistic curves are fitted to the resulting frequency data and median transition times are thus estimated for each developmental stage. The larval, pupal, and teneral adult stages are studied by semi-destructive sampling, namely, by partial removal of the seed coat and direct observation of the progress of development. Thus, the entire development is divided into 12 stages, for each of which a thermal reaction norm is plotted for the first time, visualizing the changes in the temperature-sensitivity of development from oviposition to adult emergence.

## Results

In total across all the five temperature regimens, 2444 stained embryos were mounted and inspected using fluorescence microscopy (for raw numerical data, see Additional file 1), 7248 post-dorsal-closure embryos were processed under a light microscope (Additional file 2), and 574 adult beetles were reared from first-instar larvae (Additional file 3). It was not possible to measure immature survival during any period from oviposition to pupation because some eggs were infertile, some embryos were lost during fixation or staining, and the exact initial number of larvae for the postembryonic development experiment was not known. However, prepupae showed 100% survival to adult emergence, barring one individual adult at 26 °C that failed to emerge from the seed despite successful eclosion. The actual incubation temperatures slightly deviated from the set values and are provided in Tables 1 and 2.

**Table 1:** Temperature-dependent embryonic and early larval development in *Callosobruchus maculatus*: median transition times (estimated using logistic regression analysis) and calculated durations of stages

Developmental stage	Median time of transition to next stage at five incubation temperatures, h from oviposition					Stage duration at five incubation temperatures, h				
	19.8 °C	22.7 °C	25.6 °C	28.6 °C	31.7 °C	19.8 °C	22.7 °C	25.6 °C	28.6 °C	31.7 °C
Early cleavages up to 256 nuclei	14.10	10.25	6.28	5.17	4.11	14.10	10.25	6.28	5.17	4.11
Late cleavages and germ band formation	34.37	24.21	18.52	14.15	12.42	20.27	13.96	12.24	8.98	8.32
Germ band extension	54.63	39.16	28.03	21.09	17.52	20.26	14.95	9.51	6.94	5.10
Germ band retraction	107.90	75.33	57.06	41.63	34.51	53.27	36.18	29.03	20.54	16.99
Dorsal closure	167.85	115.20	85.78	64.89	54.66	59.95	39.87	28.73	23.26	20.15

	19.7 °C	22.6 °C	25.6 °C	28.8 °C	31.6 °C	19.7 °C	22.6 °C	25.6 °C	28.8 °C	31.6 °C
Final morphogenesis	211.32	134.54	105.34	80.57	68.81	43.47	19.34	19.56	15.69	14.15
Sclerotization	235.74	157.76	118.68	89.66	78.59	24.42	23.22	13.33	9.08	9.78
Hatching	269.90	177.72	130.58	99.12	87.09	34.15	19.96	11.91	9.47	8.50
Boring into the seed	351.53	229.27	172.34	129.15	112.20	81.63	51.55	41.76	30.03	25.12

Temperature values in bold refer to actual incubation temperatures which slightly differed from the set values of 20, 23, 26, 29, and 32 °C

**Table 2:** Temperature-dependent development in *Callosobruchus maculatus* inside cowpea seeds

Developmental stage	Median stage duration with lower and upper quartiles at five rearing temperatures, d				
	20.0 °C	23.0 °C	25.9 °C	28.9 °C	31.8 °C
Larva (feeding and pupation)	38.9 (35.0–49.8)	22.6 (21.0–24.2)	17.1 (15.9–18.9)	14.0 (11.8–15.0)	12.9 (11.0–13.8)
Pupa	10.3 (9.8–10.8)	6.9 (6.0–7.0)	5.9 (5.0–6.2)	5.0 (4.2–5.3)	4.1 (3.9–5.0)
Teneral adult	6.8 (6.0–7.1)	4.5 (4.0–5.0)	3.0 (3.0–4.0)	3.1 (2.0–5.0)	4.0 (2.9–5.0)

### Embryonic and early postembryonic development

**Fig. 1:**
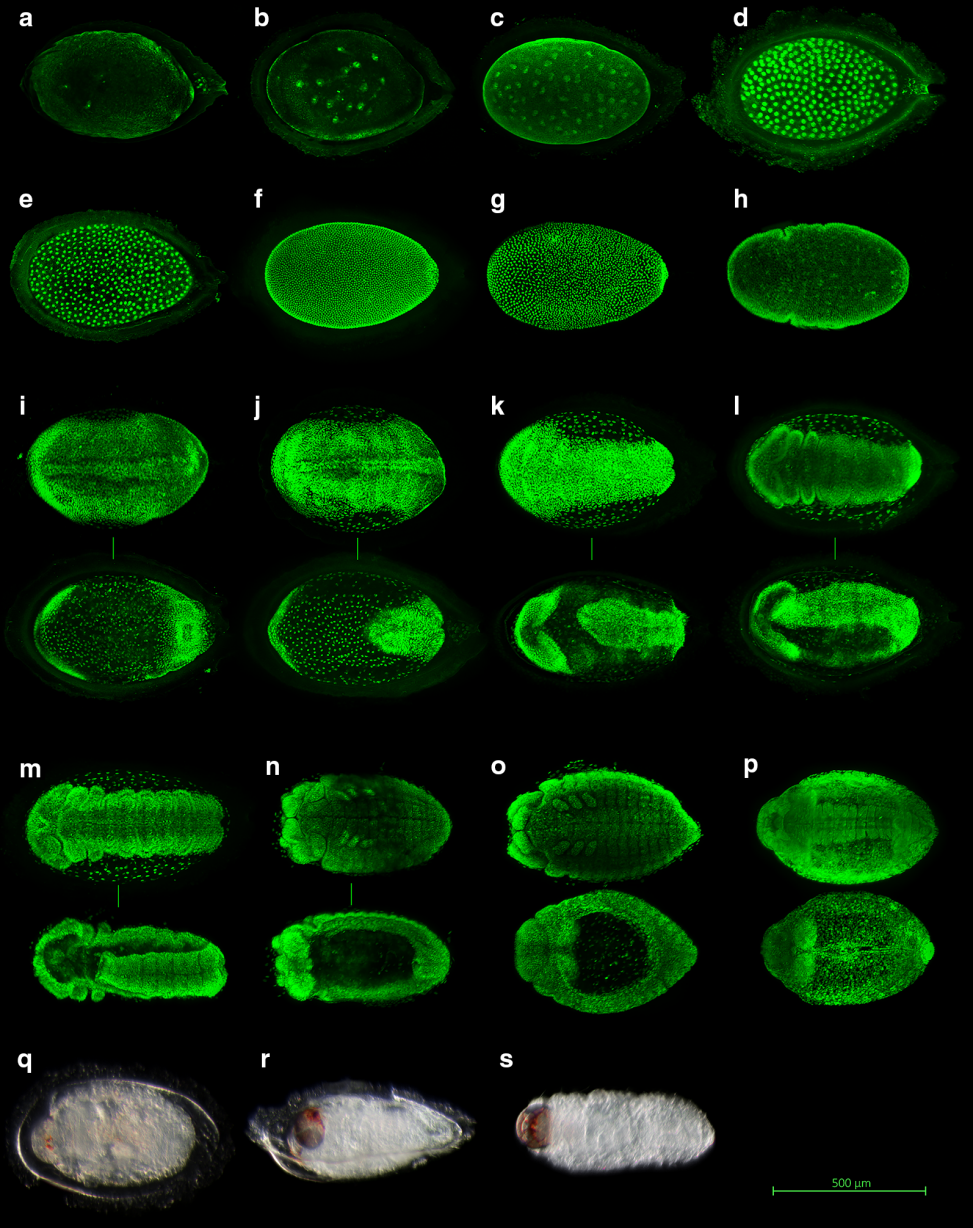
Embryonic and early larval development in *C. maculatus* as visualized with (**a**–**p**) confocal laser scanning microscopy and (**q**–**s**) light microscopy. Embryos stained with SYTOX Green fluorescent nucleic acid stain: (**a**–**f**) selected cleavages: 2, 16, 64, 256, 1024, and 4096 nuclei, respectively; (**g**) a mitotic wave during transition from 4096 to 8192 nuclei; (**h**) invagination of a transverse furrow during gastrulation; (**i**) a three-layered, segmented germ band; (**j**–**l**) germ band extension; (**m**) germ band retraction; (**n**) the end of germ band retraction and the onset of dorsal closure; (**o**–**p**) dorsal closure; in paired images (**i**–**p**), the upper and the lower parts correspond to a ventral and a dorsal view, respectively, and belong to different but same-stage embryos, except for (**n**) that shows the same embryo from both sides. Late embryos at different stages of sclerotization and hatching: (**q**) reddening of the mandibular tips, ventral view; (**r**) hatching, viewed ventrolaterally – note that the fully formed larva has rotated 180° and is now emerging through the former dorsal side of the egg; (**s**) freshly hatched first instar larva, ventral view

**Fig. 2:**
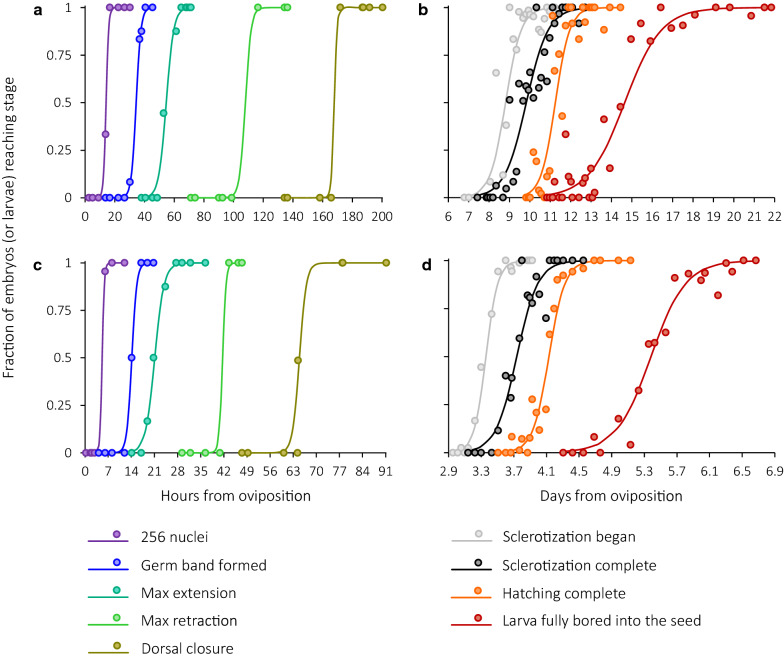
Transition from one developmental stage to the next at two incubation temperatures: (**a**, **b**) 20 °C and (**c**, **d**) 29 °C. Logistic regression curves are based on maximum penalized likelihood (**a**, **c**) or usual maximum likelihood (**b**, **d**). Each data point refers to one sample, i.e., a group of eggs laid during a 20-min period, but one and the same sample may appear on the graph more than once if it was used for plotting different curves. Logistic curves for the other three incubation temperatures can be found in Additional file 4

**Fig. 3:**
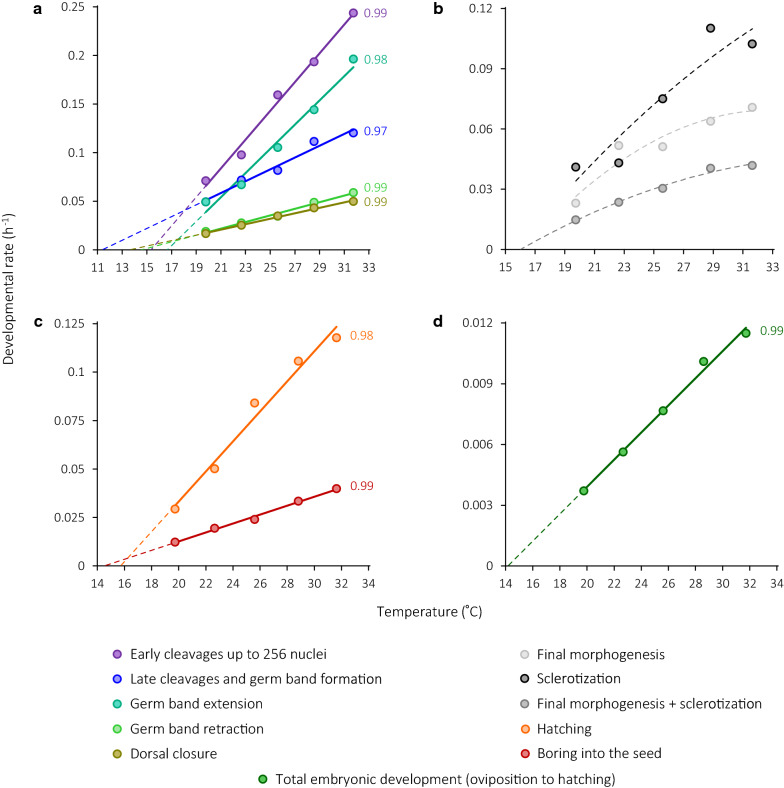
Thermal reaction norms for (**a**) embryonic stages until the completion of dorsal closure, (**b**, **c**) post-dorsal-closure embryonic stages, and (**d**) total embryonic development. Bold solid lines with *R*^2^ values shown near them are plotted based on the results of linear regression analyses, which are summarized in Table 2. Dashed lines designate either linear extrapolation beyond the studied temperature range or provisional second-order polynomials shown for illustration purposes

Eggs of *C. maculatus* are small, about 0.6 mm in length, and somewhat teardrop-shaped. Near the vegetal pole of the embryo, the chorion is extended into a short respiratory tube with conspicuous protuberances on its inner surface (best visible in Fig. 1a, d, e, j, l). The exposed surface of the egg, which later corresponds to the ventral side of the embryo, is convex and flanged, while the opposite (embryo’s dorsal) surface is flattened and clings tightly to the seed (Fig. 1r). Early embryogenesis in *C. maculatus* proceeds similarly to that in other insects: a series of initial rapid cleavage divisions take place within the yolk mass (Fig. 1a–c) but, starting from the 256-nuclei (young syncytial blastoderm) stage, all cleavages are superficial (Fig. 1d–g). The mature blastoderm is differentiated into a ventrally positioned germ band and an extraembryonic region (Fig. 1h–i). Given the stained material available, it is possible to divide this initial developmental period into two stages delineated by easily discernible developmental milestones: early cleavages up to the 256-nuclei stage and blastoderm cleavages plus germ band formation (Table 1). The germ band subsequently extends dorsally so that its anterior and posterior ends almost meet (Fig. 1j–l). The retraction that follows eventually results in the anterior and posterior ends of the germ band being positioned at the opposite poles, which marks the start of dorsal closure (Fig. 1m–n). Germ band retraction is similar in duration to the entire preceding development and to the subsequent process of dorsal closure itself (Table 1), during which the dorsal epithelial hole becomes sealed shut (Fig. 1n–p). During all the five stages mentioned, development is rather synchronous with abrupt transitions from one stage to the next (Fig. 2a, c; Additional file 4). The corresponding developmental rates show a linear relationship with incubation temperature (Fig. 3a, Table 3). The lower temperature thresholds (LTTs) vary from 11.4 to 16.7 °C with no evident unidirectional pattern (Fig. 3a, Table 3).

**Table 3:** Linear regression parameters and lower thresholds for temperature-dependent development in *C. maculatus*

Developmental stage	*a* ± SE, days^−1a^	*b* ± SE, (°C × days)^−1a^	LTT ± SE, °C
Early cleavages	− 0.2269 ± 0.02268	0.01479 ± 0.00087	15.3 ± 2.2
Late cleavages and germ band formation	− 0.0692 ± 0.01565	0.00608 ± 0.00060	11.4 ± 2.6
Germ band extension	− 0.2075 ± 0.02759	0.01245 ± 0.00106	16.7 ± 2.3
Germ band retraction	− 0.0495 ± 0.00561	0.00340 ± 0.00022	14.6 ± 2.2
Dorsal closure	− 0.0382 ± 0.00336	0.00281 ± 0.00013	13.6 ± 2.2
Hatching	− 0.1220 ± 0.01736	0.00776 ± 0.00067	15.7 ± 2.3
Total embryonic development^b^	− 0.0095 ± 0.00080	0.00067 ± 0.00003	14.2 ± 2.2
Boring into the seed	− 0.0335 ± 0.00288	0.00231 ± 0.00011	14.5 ± 2.2
Larval feeding and pupation	− 0.0793 ± 0.00199	0.00527 ± 0.00009	15.1 ± 0.4
Pupal stage	− 0.1607 ± 0.00478	0.01295 ± 0.00023	12.4 ± 0.6

^a^Regression parameters with their standard errors were determined with ordinary linear regression (one observation per temperature, Fig. 2), except for the larval feeding and pupal periods where generalized least-squares models were fitted under restricted maximum likelihood to the entire datasets of individual developmental rates (Fig. 3). ^b^Includes all of the foregoing as well as final morphogenesis and sclerotization, the thermal responses of which were found to be nonlinear

Depending on the incubation temperature, dorsal closure is completed almost or exactly halfway between oviposition and full penetration into the seed. The duration of the post-dorsal-closure development is 1.09, 0.99, 1.00, 0.99, and 1.05 times that of the preceding development at 20, 23, 26, 29, and 32 °C, respectively. The post-dorsal-closure period is divided into final morphogenesis (a stage tentatively so named that ends with the reddening of the mandibular tips), sclerotization (until full melanization of the cuticular sclerites), and hatching (Table 1, Fig. 1q–s). Developmental rates during the former two stages are quite variable (Fig. 2b, d) and, although the rates do increase with incubation temperature, the position of the data points strongly deviates from a linear relationship (*R*^2^ = 0.86 in the case of final morphogenesis and *R*^2^ = 0.88 for sclerotization). A low number of data points (one estimate per stage per temperature) prevents meaningful fitting and comparison of models. A quadratic function of the form *R* = − 0.00009*T*^2^ + 0.0073* T*—0.0918 fitted to combined developmental rates from dorsal closure until the end of sclerotization yields a provisional lower temperature threshold of 16.0 °C (Fig. 3b).

As all of the previous development occurs with the embryo’s ventral side facing away from the seed, a fully sclerotized larva has to rotate its body inside the eggshell prior to boring into the cowpea seed tissues. This rotation and subsequent perforation of the eggshell by the emerging larva is defined here as hatching (Fig. 1r). Hatching speed linearly increases with temperature, and so does the speed of boring (Fig. 3c). Of all the processes occurring under the eggshell, larval penetration into the seed is the least synchronized one (Fig. 2b, d), which is especially evident at low rearing temperatures. For example, at 20 °C, the first larvae to have completely bored into the cowpea seed were observed on the 12th day since oviposition, and yet it took another 10 days for all of the larvae to do so. The ‘true’ embryonic period in *C. maculatus* from oviposition to hatching varies from 11.2 days at 19.8 °C to 3.6 days at 31.7 °C and requires 1492 degree-hours, or 62.2 degree-days, above the threshold value of 14.2 °C.

### Larval and pupal development

**Fig. 4:**
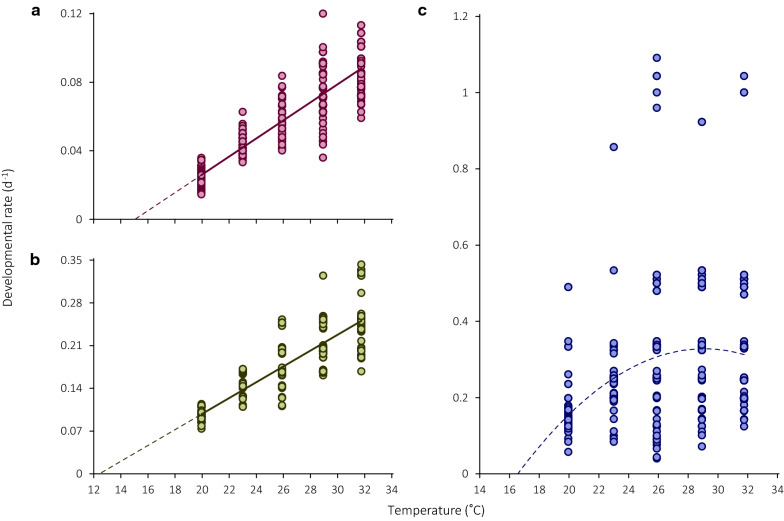
Thermal reaction norms for (**a**) larval, (**b**) pupal, and (**c**) adult teneral development in *C. maculatus*. Data points correspond to individual developmental rates. Regression lines in **a** and **b** are plotted based on GLS model parameters fit by REML. The dashed curve in **c** refers to a second-order polynomial, plotted for illustration purposes

Larval and pupal developmental rates increase with temperature in the studied range (Fig. 4a, b; Tables 2 and 3), and the effect of temperature is highly significant (larvae: *F*_1, 570_ = 3727.9, *p* < 0.00001; pupae: *F*_1, 570_ = 3311.6, *p* < 0.00001). There is no significant difference in developmental rate between males and females (larvae: *F*_1, 570_ = 0.3, *p* = 0.6; pupae: *F*_1, 570_ = 0.003, *p* > 0.9). The temperature-by-sex interaction is nonsignificant in larvae (*F*_1, 570_ = 1.0, *p* = 0.3) and significant in pupae (*F*_1, 570_ = 7.7, *p* = 0.01). Due to the negligible absolute differences, developmental data are combined across sex. Larval development in *C. maculatus*, excluding the initial stage of boring into the seed, requires 189.8 degree-days above the LTT of 15.1 °C and pupal development requires 77.2 degree-days above 12.4 °C (Table 3).

### Teneral adult development and adult body mass

Adult developmental rate during the teneral stage is significantly influenced by rearing temperature (*F*_1, 570_ = 256.9, *p* < 0.00001) but not by sex (*F*_1, 570_ = 0.003, *p* = 0.9) or its interaction with temperature (*F*_1, 570_ = 0.03, *p* > 0.9). The duration of the teneral stage is highly variable with both the minimum (0.9 days) and the maximum (25.0 days) being recorded at the same rearing temperature of 26 °C. On average, fully formed beetles spend 3 days in the seed at 26 and 28 °C and remain within the seed for longer than that in both cooler and warmer conditions (Table 2). Due to a nonlinear relationship between developmental rate and temperature, the data are approximated with a second-order polynomial of the form *R* = − 0.0021*T*^2^ + 0.1219* T* – 1.443. The corresponding quadratic curve crosses the temperature axis at 16.6 °C (Fig. 4c). There was a small fraction of adults, mostly at higher rearing temperatures, that emerged one or two days after eclosion (Fig. 4c), which may have been triggered by the presence of an artificial incision in the seed coat. These outliers were eventually retained in the final dataset as their inclusion did not affect the shape of the temperature response.

**Fig. 5:**
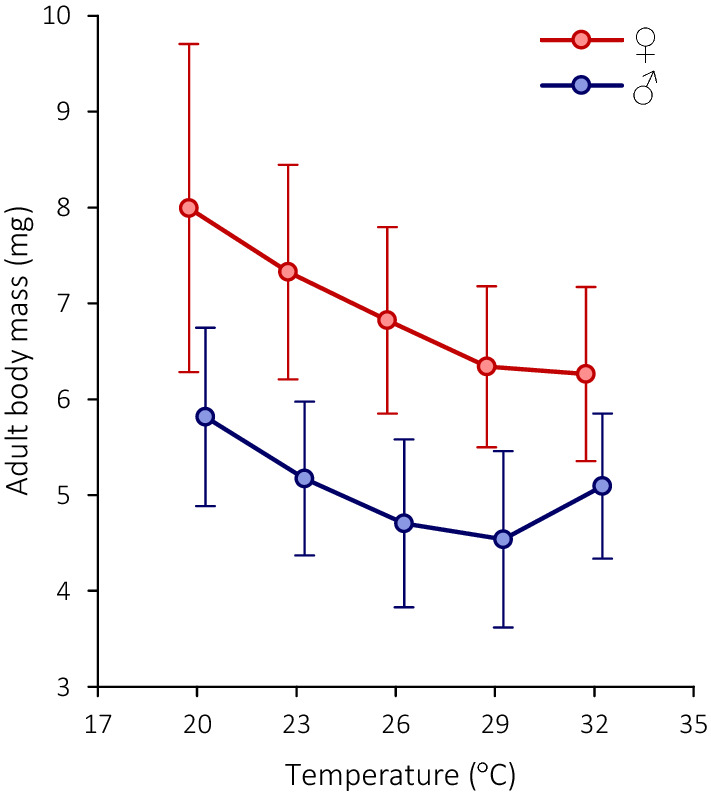
Thermal reaction norms for adult body mass in *C. maculatus* after rearing at five constant temperatures. Symbols with bars denote means ± SD and are slightly set apart along the temperature axis for clarity

Regardless of temperature, females of *C. maculatus* are significantly heavier than males (*F*_1, 570_ = 449.9, *p* < 0.00001; Fig. 5). The effect of developmental temperature on adult body mass is also highly significant (*F*_1, 570_ = 34.4, *p* < 0.00001), with heavier beetles emerging at lower temperatures (Fig. 5). The latter tendency is more pronounced in females, where the temperature-mass relationship is negative and monotonic within the studied temperature range, as opposed to a U-shaped response observed in males (Fig. 5). This difference in temperature responses between sexes is reflected in a significant temperature-by-sex interaction (*F*_1, 570_ = 10.0, *p* = 0.002).

## Discussion

This study is the first to estimate the exact durations of separate developmental stages in the cowpea seed beetle *C. maculatus* from oviposition to adult emergence at several constant, permissive temperatures. Although the life cycle of *C. maculatus* takes place by and large in one spot, first on the surface of a cowpea seed and then under that surface, and does not imply movement between different thermal environments, this beetle’s thermal phenotype varies in the course of ontogeny and different stages seem to scale disproportionately with temperature. The proximate and ultimate causes of these changes remain to be discovered, and yet the data obtained do shed some light on the developmental processes in this dangerous pest of stored legumes and widespread laboratory animal as well as lay the groundwork for comparative studies of the origin and evolution of seed beetle life cycles. While it is commonplace that development of plants and ectothermic animals becomes faster with rising temperature, and *C. maculatus* is no exception to this rule, still very little is known about the effects of temperature on particular developmental processes and on the life cycles of cryptically living species.

### Comparisons with published data

From the earliest stages onwards, there are marked differences in developmental rate between individual *C. maculatus* embryos, and development generally becomes less and less synchronous over time (Fig. 2). During fluorescent microscopy, it was not uncommon to see same-age embryos with the number of nuclei varying, e.g., from 4 to 32 or from 256 to 1024 (see also Additional file 1 for examples of samples containing both pre-blastoderm and blastoderm embryos). The coexistence of slow- and fast-cleaving embryos in a population is seldom paid attention to in the literature but seems to be a widespread phenomenon as it occurs in organisms as different as psychodid flies [28] and humans [15]. Unfortunately, destructive sampling used in this study makes it impossible to ascertain whether *C. maculatus* embryos retain the propensity for relatively slow or fast development later in the course of ontogeny and whether their development scales proportionately from one individual to the other. However, a recent study [10] on *Drosophila* finds that early embryos do maintain their individual pace of development up to advanced stages.

Early development in *C. maculatus* is quite rapid as the 2-nuclei stage is reached approximately 2.5 h from oviposition at 20 °C and after 0.5 h at 32 °C. As a comparison, the time from oviposition to first cleavage varies among insects from over 1 h in rapidly developing endoparasitic wasps [38] to over 1 d in species with long life cycles such as a stonefly [41] and a cockroach [17]. The above estimates of the timing of the first cleavage differ substantially from those reported by a previous author [68] (5–6 h at 22 °C and 2 h at 30 °C). Similarly, the *C. maculatus* colony studied here reaches the 256-nuclei stage much earlier than 12 h at 22 °C and 6 h at 30 °C [68] (cf. data in Table 1). As the handling time is minimized in both studies (for a discussion of limitations of the current experimental design, see below), this large discrepancy seems to stem from intrinsic variation between the two laboratory colonies. Another study [14] examines post-blastoderm embryogenesis in *C. maculatus* at 28 °C and, even though it only provides approximate timing of developmental events, all transition times noticeably lag behind those presented in Table 1: germ band formation occurs between 16 and 20 h after oviposition; maximum germ band extension, between 24 and 28 h; the germ band becomes fully retracted by the 52nd hour or slightly later, and dorsal closure is completed between 84 and 88 h after oviposition [14].

Thus, the *C. maculatus* colony studied here shows substantially faster embryonic development than the two previously studied laboratory colonies. In principle, the time to first cleavage can vary between animal populations [21], and stored-product pests like *C. maculatus* are all the more likely to exhibit interpopulation differences in various traits due to human-aided long-distance dispersal that is accompanied by genetic drift and local adaptation [65]. Further, multiple bottleneck events naturally result in high inbreeding rates, and inbreeding depression in *C. maculatus* is known to be manifested in decreased hatchability, impaired postembryonic survival, and prolonged development [16]. One possible explanation for the differences in embryonic developmental rates may be that the laboratory culture used in this study was not old itself and was regularly ‘refreshed’ by adding new beetles, whereas the previous studies may have used inbred material.

It seems also worthwhile to compare total developmental rates (from oviposition to adult emergence) across the six available studies, including the present one. Note that the number of studies is lower than that mentioned in the Introduction as many previous experiments were carried out with mung, chickpea, and other legumes other than *V. unguiculata* as hosts. Durations from Tables 1 and 2 were summed up, converted into rates, and plotted against weighted mean developmental temperature alongside the previously published data. Overall, data points from the present study fall well within the range of reported values (Additional file 5). This range is admittedly rather broad and can be thought of as a slow-fast continuum where the present results are closer to the slow end. In addition to possible causes of interpopulation differences mentioned above, this wide variation across studies is likely explained in part by nonidentical rearing conditions. Also, there exists a so-called ‘active form’ of *C. maculatus* distinguished by a disproportionately prolonged post-feeding period inside the cowpea as well as by reproductive diapause and high propensity for dispersal during the adult stage, but the factors inducing an increase in the frequency of this form both in the laboratory and in the field are poorly understood [7, 25, 75]. It is conceivable that different laboratory colonies may produce varying fractions of this form, which will inevitably translate into discrepancies in measured developmental rates. Thus, postembryonic developmental rates in *C. maculatus* seem to be more or less reproducible yet prone to substantial genetic and/or uncontrolled environmental variation.

On average across all experimental temperatures used in this study, males emerge from the cowpea seed at a mass of 5.07 mg and females, at 6.95 mg, which is similar to or slightly greater than in previous experiments [25, 62], indicating that final body size may be more consistent across different strains of *C. maculatus* than development time.

### Scaling of early embryogenesis across incubation temperatures

The question of whether each consecutive developmental stage takes the same proportion of total development time, regardless of temperature, has a long history in the literature on copepods and terrestrial arthropods. In the former, the constancy of the fraction of a stage in total development is termed equiproportional development [12]; in the latter, the same phenomenon is referred to as developmental rate isomorphy [26, 27, 69], and both groups include species that violate this pattern [5, 39, 52]. All of these studies mostly focus on postembryonic development while embryos, if considered at all, are never separated into stages. Recently, robustness of the timing of developmental events in relation to temperature has been addressed in a study on *Drosophila* embryos [34].

There are two approaches to testing whether development scales proportionately with temperature. The first one is, obviously, to check whether relative durations of any stages (i.e., proportions of total development) vary with temperature in any regular manner [26, 52]. However, proportions sum up to unity and as such do not represent independent observations: proportion of one stage can only increase with temperature at the expense of proportion(s) of the other stage(s). Although an appropriate method for regressing proportions does exist [5], it requires individually resolved data, which are not available for the embryonic stages of *C. maculatus*. The second approach is to compare LTT values because proportional scaling of all developmental stages with temperature is only possible when the LTT is constant throughout development [26].

With mostly indirect evidence being available half a century ago, Howe [24] envisaged the existence of many distinct LTTs during embryogenesis. The standard errors of the embryonic LTTs in *C. maculatus* are quite large for comparisons to be made, ranging from 2.2 to 2.6 °C (Table 3), but caution should be exercised with these estimates as they are based on an approximate formula [6] and, despite wide usage, the statistical properties of these standard errors are unknown [5]. Judging from my own experience with temperature-dependent developmental data, a small dataset with 4 or 5 constant temperatures, one developmental rate value per temperature, will typically yield large standard errors of about 2 °C. Thus, when the LTTs of different developmental stages are less than 4 °C apart, which seems to be rather a rule than exception, at least for immature stages of insects and mites [26], their standard errors will likely overlap. In fact, if an organism’s thermal phenotype is organized as a more or less coadapted whole, one would expect that thermal responses of various vital processes have been fine-tuned over evolutionary time to match each other as closely as possible. The use of replicates (i.e., more than one developmental rate value per temperature) will reduce the standard errors of the LTTs (cf. larval and pupal parameters in Table 3), but, even in this case, seemingly large differences in the LTT between stages may turn out to be statistically nonsignificant [57].

The unfortunate conclusion is that deviations from proportionate scaling are often minor (albeit possibly important from an organism’s perspective) and difficult to prove or reject convincingly, and so it often remains at a researcher’s discretion to decide whether the deviations are small enough to be dismissed. For example, in 11 species of *Drosophila*, embryogenesis scales evenly across incubation temperature in a nonstressful thermal range [34], although no formal tests in support of this point are carried out. To the best of my knowledge, only one work [74] has addressed the effect of temperature on the relative durations of embryonic stages in a coleopteran, namely, in the Colorado potato beetle *Leptinotarsa decemlineata* (Say); that study finds the scaling of embryogenesis with temperature to be disproportionate, yet also without any statistical analysis.

The aim of the foregoing discussion is rather to highlight the problem than to resolve it; in fact, the LTT values for different stages of *C. maculatus* in this study are obtained with different methods and their direct comparison is problematic. In any case, the LTTs of the embryonic stages vary from 11.4 to 16.7 °C, and all subsequent variation in the LTT up to adult emergence remains within this broad range (Table 3, Figs. 3 and 4). Late cleavages until germ band formation are especially notable in this regard as this stage has the minimum LTT and, over the temperature range tested, increases its proportion in early embryonic development: 0.12 at 20 and 23 °C, 0.14 at 26 and 29 °C, and 0.15 at 32 °C (the completion of dorsal closure being considered as 1). This increase is counterbalanced by variation in the proportion of germ band extension from 0.12 at 20 °C to 0.09 at 32 °C.

Multispecies datasets may help in better understanding the scaling of embryogenesis with temperature: if a similar scaling pattern recurs from species to species, it is less likely to be happenstance. So far, such information is only available for *Drosophila*, where embryogenesis is claimed to scale evenly across 11 species [34]. However, the thermal reaction norms for development time of tropical and temperate *Drosophila* species markedly intersect in the nonstressful temperature range (Fig. 5 in [34]), which clearly indicates to the contrary: there should be a significant temperature by species interaction and thus a violation of rate isomorphy. Disproportionate scaling can also be found within species as, e.g., different geographical populations of the ascidian chordate *Ciona* seem to have widely different LTTs for total embryonic development [72].

To summarize, research on temperature-dependent embryogenesis is still itself in its early development and it would require cooperative efforts among thermal ecologists, developmental biologists, and biostatisticians to properly analyze the scaling of different embryonic and postembryonic processes across ecological factors, populations, and species.

### Larval and pupal development

The processes of hatching, boring into the cowpea, and feeding (including the actually nonfeeding prepupal stage) have similar LTTs varying from 14.5 to 15.7 °C (Table 3). Seed beetles have four larval instars separated by molts [30] but their identification in *C. maculatus* requires destruction of infested cowpea seeds and was beyond the scope of this work, which is mainly focused on embryogenesis. The pupal LTT (12.4 °C) is lower than in the previous stages, which seems to violate the rate isomoprhy assumption, and the pupa:feeding duration ratio increases from 0.24 at 20 °C to 0.30 at 23 °C, 0.33 at 26 °C, and 0.35 at 29 and 32 °C. The abovementioned LTTs are similar to those in the other two bruchines for which such data are available: 14.4 °C in larvae vs. 12.5 °C in pupae of *C. rhodesianus* (calculated by linear extrapolation from data in Table V in [25]), and 15.7 °C in larvae vs. 12.6 °C in prepupae + pupae of *B. pisorum* (calculated by linear extrapolation from data in Tables 1 and 2 in [59]). Based on just three species, it would be premature to draw definitive conclusions about the evolutionary conservatism of the larval and especially pupal LTT, but the remarkable agreement between different studies is worth attention. Disproportional scaling of the pupal duration with temperature in different seed beetle species may indicate phylogenetic inertia or an ecophysiological adaptation, or both. Also, as noted above, the laboratory colony studied may have contained a significant fraction of the ‘active form’ of *C. maculatus* that has a prolonged post-feeding period [7]. No attempt was made from the beginning to distinguish the two forms of the beetle, preventing further elaboration of this idea. Nevertheless, the very existence of such polyphenism is another example of how developmental durations can differ disproportionately between alternative phenotypes.

### Nonlinear developmental responses to temperature

By definition, a nonlinear response implies that the stage in question does not scale uniformly with temperature. There are two periods in *C. maculatus* ontogeny during which developmental rates depend on temperature in the nonstressful thermal range in a strongly nonlinear fashion. Although this may well be a coincidence, the main process during both these periods is sclerotization, i.e., hardening and darkening of the integument, either at the end of embryogenesis (Fig. 3b) or at the end of postembryonic immature development (Fig. 4c), and the LTT during these periods is also similar (16.0 and 16.6 °C, respectively). As sclerotization is a very complex process, many details of which are poorly understood, it may be presumed that different biochemical reactions during this process have somewhat discordant temperature responses and/or are strongly sensitive to other extrinsic or intrinsic factors.

The temperature-dependence of *C. maculatus* adult body mass at emergence is also nonlinear (Fig. 5) and exhibits a pattern widely known as the ‘temperature-size rule’ [3], i.e., a progressively larger body size at lower developmental temperatures. The same negative relationship is also observed in all other studies that examine the effect of rearing temperature on body mass in this beetle [9, 25, 62]. The ‘temperature-size rule’ is not universal among ectotherms but small-sized, multivoltine terrestrial arthropods are more likely to conform to it [23], and this is exactly the case with *C. maculatus*. It is also of note that males and females respond slightly differently to rearing temperature such that the male response is more curvilinear (Fig. 5), which remarkably resembles the results of Stillwell et al. [62] and indicates that body mass per se and its plasticity are well reproducible in *C. maculatus*.

### Limitations imposed by the experimental design

In principle, all biological rates, including developmental rate, are known to increase with temperature monotonically and quasi-linearly over a more or less broad nonstressful thermal range. Deviations from this quasi-linear relationship may arise from an imperfect match between that range with actual experimental temperatures and/or from various confounding factors such as imprecise control and measurement of temperature, observational errors in determining stage transition times, large intrapopulational variation, unnoticed differences in humidity or other rearing conditions, etc. While every effort was made in this study to minimize these sources of ‘noise’, it is extremely difficult, if ever possible, to eliminate them completely.

Embryonic development was assumed to start at the moment of collection of eggs, which were no older than 20 min (at 24 °C) since oviposition. It took additional 10–15 min at room temperature to cut the embryos off the seeds and soak them in bleach prior to fixation and then development was assumed to have stopped. These sources of observational error could have affected the estimated development times, but likely negligibly. The number of embryos per group was not controlled; however, as *C. maculatus* eggs are laid singly and the species is not gregarious, it seems unlikely that neighboring embryos could somehow affect each other’s development. Another source of uncertainty, specific to this study, is that durations of embryonic stages were obtained by subtraction of one estimated transition time from the other with both estimates being prone to measurement error. Certainly, observations in vivo, especially unintermittent ones, can provide more robust estimates of transition times, but this method is not without its own challenges [34] and difficult to apply to colorless, nontransparent or firmly attached embryos.

Semidestructive sampling of postembryonic stages may have affected development time but this seems unlikely, at least for larvae and pupae, given the 100% survival rates and the similarity of estimated total development times to previous measurements from the literature (Additional file 5).

The five experimental temperature regimens used in this study were chosen after reviewing the published evidence (Additional file 5) and were anticipated to fit within the linear region of the thermal reaction norm. Visual inspection of thermal reaction norms for blastoderm cleavages (Fig. 3a), hatching (Fig. 3c), and total embryogenesis (Fig. 3d) suggests that data points at 32 °C deviate from a linear relationship, possibly indicative of a nonlinear response – however, as no data is available for still higher temperatures, this deviation cannot be distinguished from experimental ‘noise’ and it seems best to stick to more conservative estimates provided by a simpler (i.e., linear) model. As even slight shifts in the position of data points may significantly affect the LTT value, due to the extrapolated nature of the latter, the credibility of the LTT estimate is highest when *R*^2^ is as close to 1 as possible.

## Conclusions

This communication addresses temperature-dependent immature development in *C. maculatus*, a dangerous cosmopolitan pest of stored legumes and one of the most widely used laboratory animals. At least during certain developmental periods, the thermal phenotype of *C. maculatus* scales unevenly over the permissive temperature range. Disproportionate changes in developmental durations with temperature may have important implications, e.g., when study design requires a high degree of synchronization among experimental embryos or when the aim of the study is to predict the occurrence of particular stages in the field, as well as in any other cases when development times need to be estimated with good precision.

The evolutionary pathways that have led to the wide diversity of development times and that make an organism develop precisely at the rate its ancestors did, with due correction for temperature and other environmental variables, remain largely unknown. Furthermore, it is rare that such problems are put forward at all. The current rise of eco-evo-devo gives hope that the patterns and mechanisms behind the evolution of development time will start to come to light.

## Materials and methods

### Stock culture

The laboratory colony was started in 2017 by purchasing a batch of dry black-eyed cowpea *Vigna unguiculata* (L.) Walp., which was unmistakably infested with *C. maculatus*, at a market in St. Petersburg, Russia (the cowpea itself was of Central Asian origin). The seeds were put in sealed 2-L plastic containers with ventilation holes in the lid and stored at a temperature of 24–25 °C and 75% relative humidity. Both in the stock culture and in all of the experimental trials mentioned below, this humidity level was maintained with the use of saturated sodium chloride solutions. No water or supplementary food was provided to the adult beetles. A new batch of cowpea, always of the black-eyed variety, was bought every several months and *C. maculatus* beetles, if any happened to be reared from the newly acquired seeds, were added to the culture to maintain genetic diversity. The initial number of founder individuals was not known but the laboratory colony rose to many thousands of individuals after half a year. Population density was not precisely controlled. As soon as cowpea deteriorated, beetles were transferred to new 2-L containers filled with fresh seeds (several hundreds of parental individuals per container). To divert the excessive moisture and thus slow down the deterioration of the seeds, 5–6 cardboard tubes per container were inserted in the mass of cowpea; this also facilitated collection because newly emerged beetles tended to gather on the surfaces of the tubes. Two months prior to the experiments, the laboratory colony was divided into two halves that were subsequently kept under two antiphase 12 h/12 h light/dark cycles, which allowed retrieval of freshly laid eggs at any time of day and night (it was found out empirically that oviposition rate was reduced right before and shortly after the dark phase).

### General experimental design

The experiments described below were carried out on numerous occasions from July 2019 till March 2020. To ensure that large amounts of freshly laid eggs could be collected simultaneously, main experimental work was timed to coincide with cyclic mass emergence of adult beetles in the stock culture. On each collection day, approximately 300–600 beetles were confined in an individual 250-ml plastic container. Care was taken to choose the suitable half of the stock culture that would have remained under the light phase for several hours at least and so would have allowed continuous collection of eggs. A 4-cm Petri dish with 15 intact cowpea seeds was put in the 250-ml container with beetles and the container was returned to the environmental chamber where the rest of the stock culture was kept at a constant temperature of 24 °C. The seeds were quickly replaced with new ones after 20 min, which was found to be a convenient period as it introduced only a minor error in the measurement of development time and prevented female beetles from laying an unmanageably large amount of eggs. This collection procedure was then repeated as many times as needed. After collection, the beetles were either discarded or released back into the culture containers. Each harvested group of 15 cowpeas with freshly laid eggs cemented to them was immediately transferred to a 4 cm^3^ plastic cup and assigned to one of the five constant temperature regimens: 20, 23, 26, 29 or 32 °C, all in constant darkness and at 75% relative humidity. The number of eggs per group was not known until fixation and so the temperature assignment was random with regard to sample size. Temperature in the environmental chambers was maintained accurate to ± 0.1–0.5 °C via a software-controlled balance of heating and cooling (RLDataView 1.03; Research Laboratory of Design Automation, Taganrog, Russia) and automatically recorded every 10 s.

### Embryonic development until dorsal closure

Egg-laden cowpeas were incubated at constant temperatures for various lengths of time since oviposition. A group of embryos that were simultaneously collected, incubated together in the same cup, and simultaneously fixed is referred to as a sample throughout the text. Initially, fixation was done at 10–15-h intervals to gain an overview of the developmental timeline. When the durations of embryonic stages of interest could be estimated to a good approximation, the transitional periods between these stages were sampled at a higher temporal resolution. As only a limited number of eggs could be collected daily and only a limited number of samples could be fixed during the working hours, fixation was done according to a schedule and there were no replicates for any sample of a given age at a given temperature. Thus, embryo samples fixed a few hours apart after egg laying could have actually been obtained from different generations of beetles and separated by weeks or months (Additional file 1), partially compensating for the lack of replication.

The fixation and staining protocol was largely based on the existing protocols developed for *C. maculatus* [14] and *Dermestes maculatus* [73]. Eggs were removed from the seeds by cutting the underlying seed coat with a sharp ophthalmological scalpel and transferred to a nylon mesh strainer. The strainer was then dipped for 4 min in 50% commercial bleach and stirred occasionally, which was followed by three washings under the cold tap and additional rinsing in 0.4% NaCl solution with Triton X-100 (250 µl/l). The rinsed embryos were transferred with a fine paint brush to an Eppendorf tube and fixed for 30 min in a 1:1 mix of 5% paraformaldehyde and heptane on an orbital shaker set to 220 rpm and 25 °C. After fixation, the lower, aqueous phase of the fixative solution was removed and 100% methanol (chilled to -20 °C) was added. The tube was capped and shaken vigorously for 20 s, which usually resulted in most of the embryos sinking to the bottom. If a significant fraction of embryos was still floating, more methanol was added and/or the tube was shaken again. After shaking, all of the solution was removed and embryos were washed three times in chilled 100% methanol and then stored in methanol at -20 °C.

Before staining, the embryos were rehydrated using a graded series of methanol/PBST and rinsed twice in PBST. Eggshells and lingering fragments of seed coat were removed manually in PBST by using two sharpened entomological pins. Eggshell removal was found to be unnecessary for developmental staging (to the degree of detail required in the present study), except in some advanced embryos, but was helpful in obtaining clearer images of post-gastrulation stages. For staging, embryos were incubated with 1:3000 SYTOX Green (Invitrogen) at room temperature for 40 min in the dark, followed by three washings with PBST and mounted in a 75% glycerol solution in PBST. A phosphate-free buffer (SSC) was also tested instead of PBST during pilot trials but the quality of staining was visually identical. Embryos were imaged using a 10 × objective on a Leica TCS SP5 confocal laser scanning microscope. Developmental stages were identified by comparing the observed embryos with drawings and photographs from previous studies on *C. maculatus* [14] and *Acanthoscelides obtectus* (Say) [29, 44]. As embryo orientation was important in the identification of maximum extension and retraction of the germ band as well as dorsal closure, some samples were re-mounted, sometimes repeatedly, until all of the embryos were observed in the desired position.

### Late embryonic and early larval development

As with early embryos, incubation at five constant temperatures was interrupted at scheduled times to assess the developmental stage reached. However, advanced embryos could be staged visually, owing to the presence of hardened and darkened cuticle. Embryos in each sample were dissected under a Leica MZ16 stereo microscope by using an entomological pin that was sharpened to resemble a cutting blade. The procedure was less time-consuming and allowed partial replication: i.e., there was sometimes more than one sample per given length of time from oviposition, rounded to the nearest hour (Additional file 2). These replicates were eventually pooled together as the results proved to be reproducible over time (for simplicity, the result of pooling is also called a sample). Embryonic development was assumed to end with hatching but, as first-instar larvae never appeared outside the eggshell, the process of boring into the cowpea seed was also tracked by destructive sampling as described above.

### Development inside the cowpea

To obtain eggs, several hundreds of beetles from the stock culture were placed for 24 h in a separate 250-ml container filled one-third full with intact cowpea. Two such collections took place on October 2 and 3 and one more, on October 23. Cowpea with eggs was incubated at 24 °C. On the 6th day of incubation, the seeds were checked for the signs of boring (white dust filling the abandoned eggshells) and the excessive larvae were culled with a needle so that only one or two larvae per seed remained. Later, occasional overlooked larvae were allowed to complete development because the large size of the seeds made competition for food unlikely. Cowpeas infested with *C. maculatus* larvae were randomly allocated among the five experimental temperatures. The seeds were monitored every third day for the presence of semi-translucent circular ‘windows’ in the seed coat. Once a ‘window’ was noticed, further observations were made on a daily basis. The seed coat was carefully incised with an ophthalmological scalpel so that the prepupa could be observed directly through a small hole in the seed coat. If the incision happened to occur before the prepupal stage and the larva was still active enough to mend the injure in the seed coat with its oral secretion, the orifice thus closed had to be reopened with a scalpel after a day or two. Preliminary experiments showed that such intervention did not affect either survival rate or development time. Pupation, adult eclosion, and emergence from the seed were recorded daily. Overlooked pupae and adults were discarded. Adults were weighed on a Discovery DV215CD electronic balance with 0.01 mg precision (Ohaus Corporation, USA) on the day of eclosion. Sex was confirmed by dissection.

### Statistical analyses

Statistical analyses were carried out in R version 4.0.0 with RStudio [53, 54]. The proportion *P* of embryos or early larvae completing the stage in question was calculated in each sample and then plotted over time since oviposition. For each developmental stage, the transition from 0 (all individuals are at the preceding stage) to 1 (all individuals have reached the next stage) was approximated with logistic regression using the brglm package [33] and the median transition time was determined at *P* = 0.5. Small sample sizes and synchronous development before dorsal closure resulted in complete or quasi-complete separation of *P* by time since oviposition (i.e., there were zeros and unities with few or no intermediate values), and so maximum penalized likelihood was used to estimate logistic regression parameters. For late embryonic and early larval development, usual maximum likelihood logistic regression was employed. Median durations *D* of each developmental stage were then calculated as a difference between two consecutive transitions. The durations (in hours) were converted into rates *R* = 1/*D* and plotted against temperature. If the rate-temperature relationship was linear (*R*^2^ of 0.97 and higher), it was approximated with a simple linear regression equation of the form *R* = *a* + *bT* [6]. The intercept (*a*) and slope (*b*) were then used to calculate the lower temperature threshold (LTT) for each developmental stage as -*a*/*b*. The standard error of LTT was calculated according to an approximate formula provided by Campbell et al. [6]. The sum of degree-hours or degree-days was calculated as 1/*b*. Markedly nonlinear developmental responses to temperature were provisionally approximated with second-order polynomials.

Larval, pupal, and teneral adult development rates were calculated for each individual as inverse durations of the corresponding stages (days^−1^). The effects of temperature and sex on developmental rate and body mass were analyzed using the generalized least-squares (GLS) method under restricted maximum likelihood with different variances for each combination of factors [50]. Analyses were performed using the gls() function in the nlme package [51]. Significance of differences was determined with *F*-tests based on type I (sequential) sum of squares. Model assumptions of homoscedasticity, linearity, and normality of residuals were verified by inspection of raw and standardized residuals plots. As for the previous developmental stages, linear regression parameters and lower temperature thresholds were calculated where possible; otherwise, second-order polynomials were plotted over developmental data.

## Supplementary information

**Additional file 1.** Raw data for early embryonic development until dorsal closure.

**Additional file 2.** Raw data for late embryonic and early larval development.

**Additional file 3.** Raw data for postembryonic development.

**Additional file 4.** Logistic regression curves showing transition of *C. maculatus* embryos from one stage to the next (an extended version of Fig. 2, all designations as in Fig. 2).

**Additional file 5.** Total (oviposition to adult emergence) developmental rates in *C. maculatus* at various constant temperatures, data from different studies.

## Data Availability

All data generated or analyzed during this study are included in this published article and its additional files.

## Abbreviations

GLS: Generalized least squares
LTT: Lower temperature threshold
PBST: Phosphate-buffered saline with Tween 20
REML: Restricted maximum likelihood
SD: Standard deviation
SE: Standard error
SSC: Saline-sodium citrate
