# Supplementary material for: Stagewise resolution of temperature-dependent embryonic and postembryonic development in the cowpea seed beetle Callosobruchus maculatus (F.)
Source: BMC Ecol. 2020 Sep 11;20:50. doi: 10.1186/s12898-020-00318-2 (PMC7488527; doi:10.1186/s12898-020-00318-2)
Supplement: Supplementary file 4 — Additional file 4. Logistic regression curves showing transition of C. maculatus embryos from one stage to the next (an extended version of Fig. 2, all designations as in Fig. 2). [file 12898_2020_318_MOESM4_ESM.pdf]

Additional supporting information for

Kutcherov D.

Stagewise resolution of temperature-dependent embryonic and postembryonic development in the cowpea seed beetle *Callosobruchus maculatus*

Additional file 4:
Logistic regression curves showing transition of *C. maculatus* embryos from one stage to the next
(an extended version of Fig. 2, all designations as in Fig. 2)

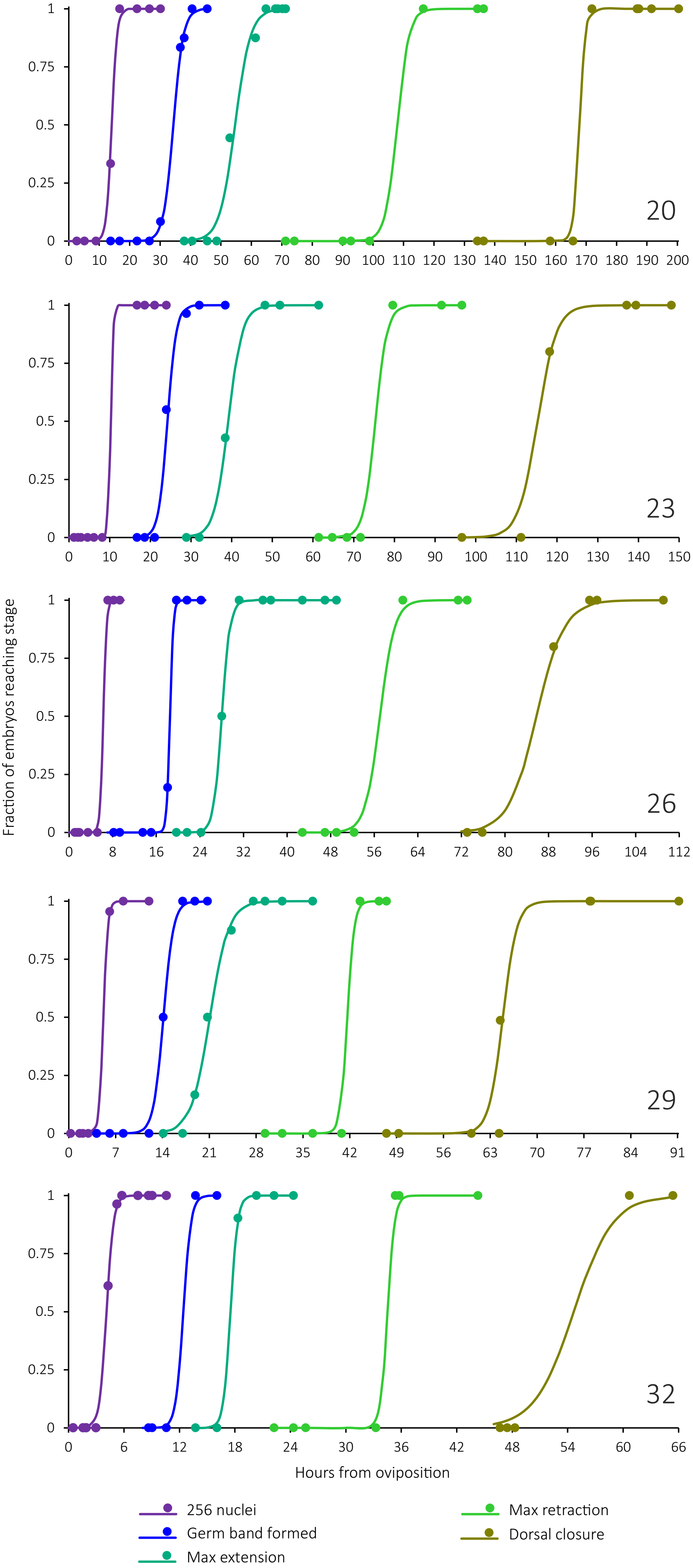

20
23
26
29
32
Fraction of embryos reaching stage
Hours from oviposition
256 nuclei
Germ band formed
Max extension
Max retraction
Dorsal closure

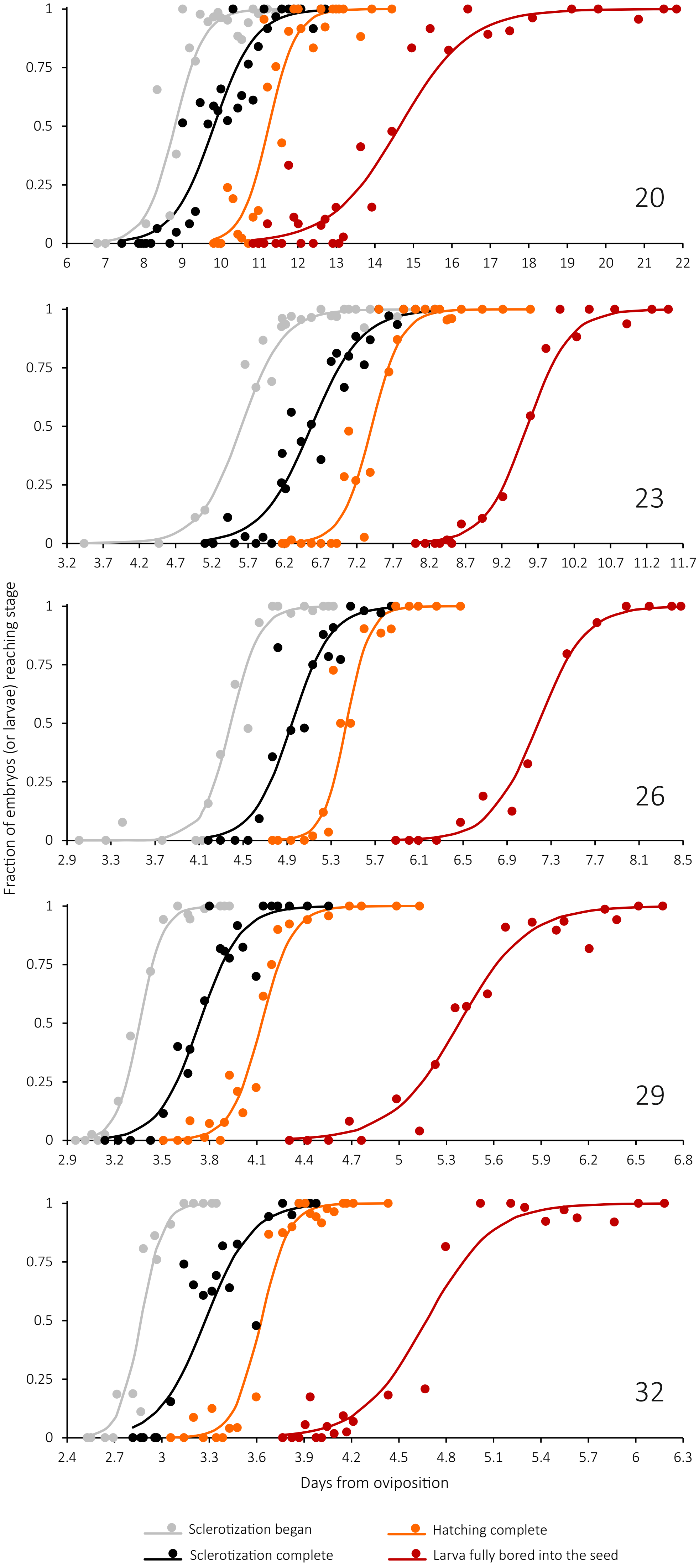
20
23
26
29
32
Fraction of embryos (or larvae) reaching stage
Days from oviposition
Sclerotization began
Sclerotization complete
Hatching complete
Larva fully bored into the seed
