# Supplementary material for: Stagewise resolution of temperature-dependent embryonic and postembryonic development in the cowpea seed beetle Callosobruchus maculatus (F.)
Source: BMC Ecol. 2020 Sep 11;20:50. doi: 10.1186/s12898-020-00318-2 (PMC7488527; doi:10.1186/s12898-020-00318-2)

Additional supporting information for

Kutcherov D.

Stagewise resolution of temperature-dependent embryonic and postembryonic development in the cowpea seed beetle *Callosobruchus maculatus*

Additional file 5: Total (oviposition to adult emergence) developmental rates in *C. maculatus* at various constant temperatures, data from different studies

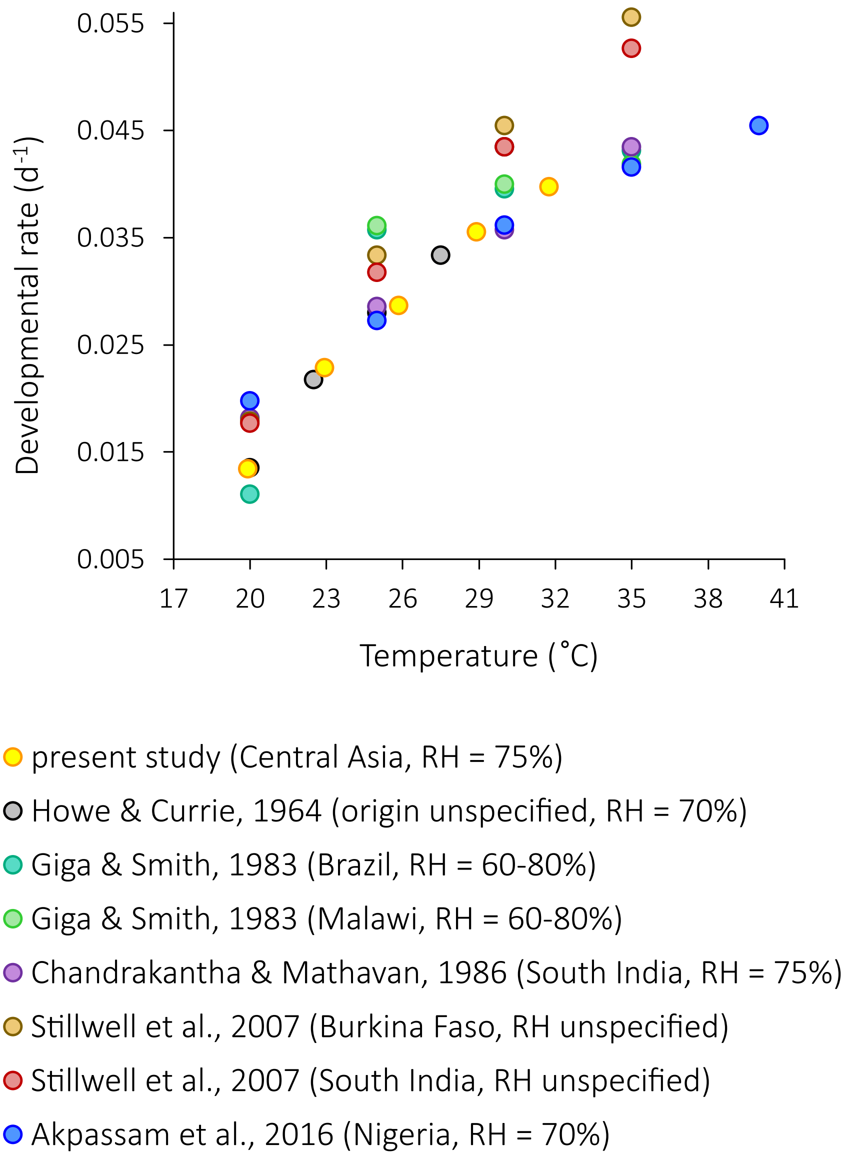

Supplement: Supplementary file 5 — Additional file 5. Total (oviposition to adult emergence) developmental rates in C. maculatus at various constant temperatures, data from different studies. [file 12898_2020_318_MOESM5_ESM.pdf]
